# Evaluating protective and therapeutic effects of alpha-lipoic acid on cisplatin-induced ototoxicity

**DOI:** 10.1038/s41419-018-0888-z

**Published:** 2018-08-01

**Authors:** Kyung-Hee Kim, Byeonghyeon Lee, Ye-Ri Kim, Min-A Kim, Nari Ryu, Da Jung Jung, Un-Kyung Kim, Jeong-In Baek, Kyu-Yup Lee

**Affiliations:** 1 Department of Biology, College of Natural Sciences, Kyungpook National University, Daegu, 41566 Republic of Korea; 2 School of Life Sciences, BK21 Plus KNU Creative BioResearch Group, Kyungpook National University, Daegu, 41566 Republic of Korea; 3 Department of Otorhinolaryngology—Head and Neck Surgery, School of Medicine, Kyungpook National University, Daegu, 41944 Republic of Korea; 4 Department of Aroma-Applied Industry, Daegu Haany University, Gyeongsan, 38610 Republic of Korea

## Abstract

Cisplatin, a small platinum-containing molecule, is a widely used, highly effective anticancer drug. However, severe side effects have been found in cancer patients treated with cisplatin, including nephrotoxicity, neurotoxicity, and ototoxicity. These cisplatin-induced side effects can have a major impact on patient quality of life, including social development problems in pediatric patients that develop hearing loss. Previous studies have suggested that the major cause of cisplatin-induced ototoxicity is abnormal accumulation of reactive oxygen species (ROS) and oxidative stress. Alpha-lipoic acid (ALA), one of the most effective antioxidants, is known to be involved in the cellular antioxidant system and may have a protective effect on cisplatin-induced ototoxicity. However, the therapeutic effect of ALA on damaged hearing function and its detailed mechanism of action are not fully understood. This study focused on determining whether ALA has a potential as a protective and/or therapeutic agent for cisplatin-induced ototoxicity. Histological and physiological analyses were performed using cisplatin-treated mouse cochlea and HEI-OC1 culture cells in pre- and post-treatment with ALA in vitro and in vivo. We found that ALA contributes to protecting mitochondrial function by preventing ROS accumulation and inhibiting apoptotic cell death. Importantly, post-treatment with ALA consistently showed an almost equal restorative effect to pretreatment, in vitro and in vivo, supporting the possible use of ALA as a therapeutic agent for cisplatin-induced ototoxicity. This study is the first report on a strong therapeutic potential of ALA to rescue ototoxic hearing loss caused by cisplatin, and our data provide key evidence that ALA may act as a reducing agent for glutathione disulfide to increase glutathione levels on behalf of glutathione reductase. This result was consistent in both cultured cells and the mouse model, which improves the clinical value of ALA for therapy of cisplatin-induced ototoxicity.

## Introduction

Since the discovery of its anticancer properties in the 1960s, cisplatin (*cis*-diamminedichloroplatinum II) has been widely used as an effective chemotherapeutic drug for a number of solid tumors, such as those found in the ovaries, lung, and bladder^1^. Cisplatin enters the cell by diffusion or a transporter and becomes hydrated, releasing its chloride ions due to lower chloride concentrations inside the cell. The hydrated form of cisplatin cross-links with nucleotides of nuclear and mitochondrial DNA to form adducts, inhibiting the indefinite replication of cancer cells^1,2^. However, clinical treatment with cisplatin is limited by adverse side effects in normal tissues, which results in nephrotoxicity, neurotoxicity, and ototoxicity during the administration of chemotherapy^2,3^.

Cisplatin-induced ototoxicity can lead to bilateral, progressive, dose-dependent, and irreversible sensorineural hearing loss and is particularly serious in pediatric populations^4^. Hearing loss can significantly hinder a patient’s quality of life, especially influencing social development such as language learning and speech in pediatric patients^5^. Although the underlying molecular mechanisms by which cisplatin causes ototoxicity is not fully understood, previous studies have demonstrated that cisplatin activates the death receptor pathway, endoplasmic reticulum-stress pathway, and mitochondrial reactive oxygen species (ROS)-generating pathway in normal cells, eventually leading to cell death^6–8^. The generation of excessive ROS is considered to be the major cause of cisplatin-induced ototoxicity, as well as the direct attack of cisplatin against DNA^8^. In particular, it has been suggested that the organ of Corti, spiral ganglion, and lateral wall in the cochlea are the primary targets of cisplatin for robust ROS generation^9,10^.

Alpha-lipoic acid (ALA), also known as thioctic acid or 1,2-dithiolane-3-pentanoic acid (C_8_H_14_O_2_S_2_), is an essential cofactor in mitochondrial dehydrogenase reactions, soluble in water and lipid, and widely distributed in the cellular membrane, cytosol, and extracellular space^11^. The protective action of ALA was first reported in 1959 by Rosenberg and Culik, who discovered its role in reducing symptoms of scurvy due to vitamin C and E deficiencies^12^. Later, its ability to regenerate endogenous antioxidants in the body, including vitamins C and E and intracellular reduced glutathione (GSH), was identified^11,13^. Recently, ALA has gained considerable attention as a universal antioxidant following discovery of its role, together with its reduced form dihydrolipoic acid as a redox couple, in free radical quenching, metal chelation, and antioxidant recycling^14,15^.

While cisplatin chemotherapy is essential for cancer patients, previous studies have only focused on the protective pretreatment effects of ALA. These studies demonstrated that major cisplatin-induced side effects including nephrotoxicity and neurotoxicity were effectively prevented by ALA preadministration in animal models, as well as hearing loss^16–19^. The most common underlying mechanism suggested by these studies was that ALA protect intracellular redox system as a powerful antioxidant, finally inhibiting cisplatin-induced apoptosis. However, it is highly valuable to develop effective therapeutic agents that have restoration ability against cisplatin-induced cytotoxicity, so that we overcome severe side effects of cisplatin. To our knowledge, this is the first study to compare the protective and alleviative effects of ALA treatment before and after cisplatin to evaluate the possible use of ALA as a therapeutic agent. We investigated the effects of ALA on cisplatin-induced hearing loss in a mouse model and speculated about the underlying mechanism of antiapoptotic pathways activated by ALA to prevent cisplatin-induced ototoxicity in auditory cell lines in vitro.

## Results

### ALA protects hearing ability and inhibits sensory cell death from cisplatin-induced ototoxicity

**Fig. 1:**
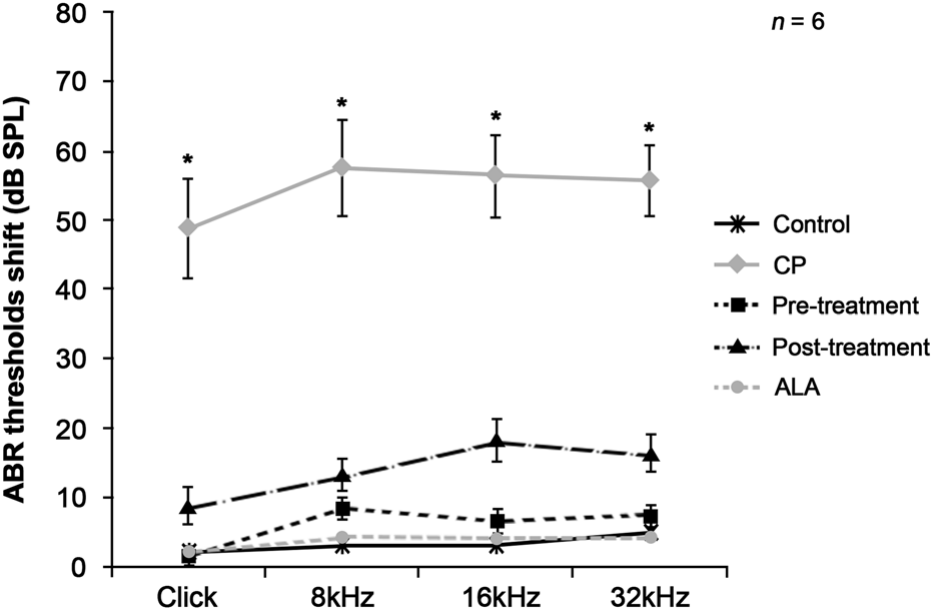
Evaluation of hearing ability in cisplatin-injected mice receiving pre- or post-treatment with ALA. Changes in ABR threshold using transient click and tone-burst (8, 16, and 32 kHz) stimuli were compared among five animal groups to evaluate hearing ability. Results are shown as the means ± SEM (*n* *=* 6 for each group). *p < 0.05, compared with CP group. ABR auditory brainstem response, ALA alpha-lipoic acid, CP cisplatin

The protective and alleviative effects of ALA on cisplatin-induced ototoxicity were examined using a mouse model. For the control and ALA groups, changes in auditory brainstem response (ABR) threshold were less than 5 dB in the entire frequency range including the click sound, indicating that ALA treatment in normal mice does not affect hearing ability; the cisplatin group showed significant changes in ABR threshold, confirming that cisplatin treatment leads to significant hearing loss (Fig. 1). Pretreatment with ALA almost completely protected hearing ability (5–10 dB change in ABR threshold), demonstrating the protective effect of ALA against cisplatin-induced hearing loss. The most interesting result was the alleviative effect of ALA, as shown by the ALA post-treatment group. Although a small difference in ABR threshold changes was observed between the ALA pre- and post-treatment groups (5–10 dB), post-treatment with ALA mostly alleviated hearing loss after cisplatin administration. This finding indicates the great potential of ALA as a therapeutic drug.

**Fig. 2:**
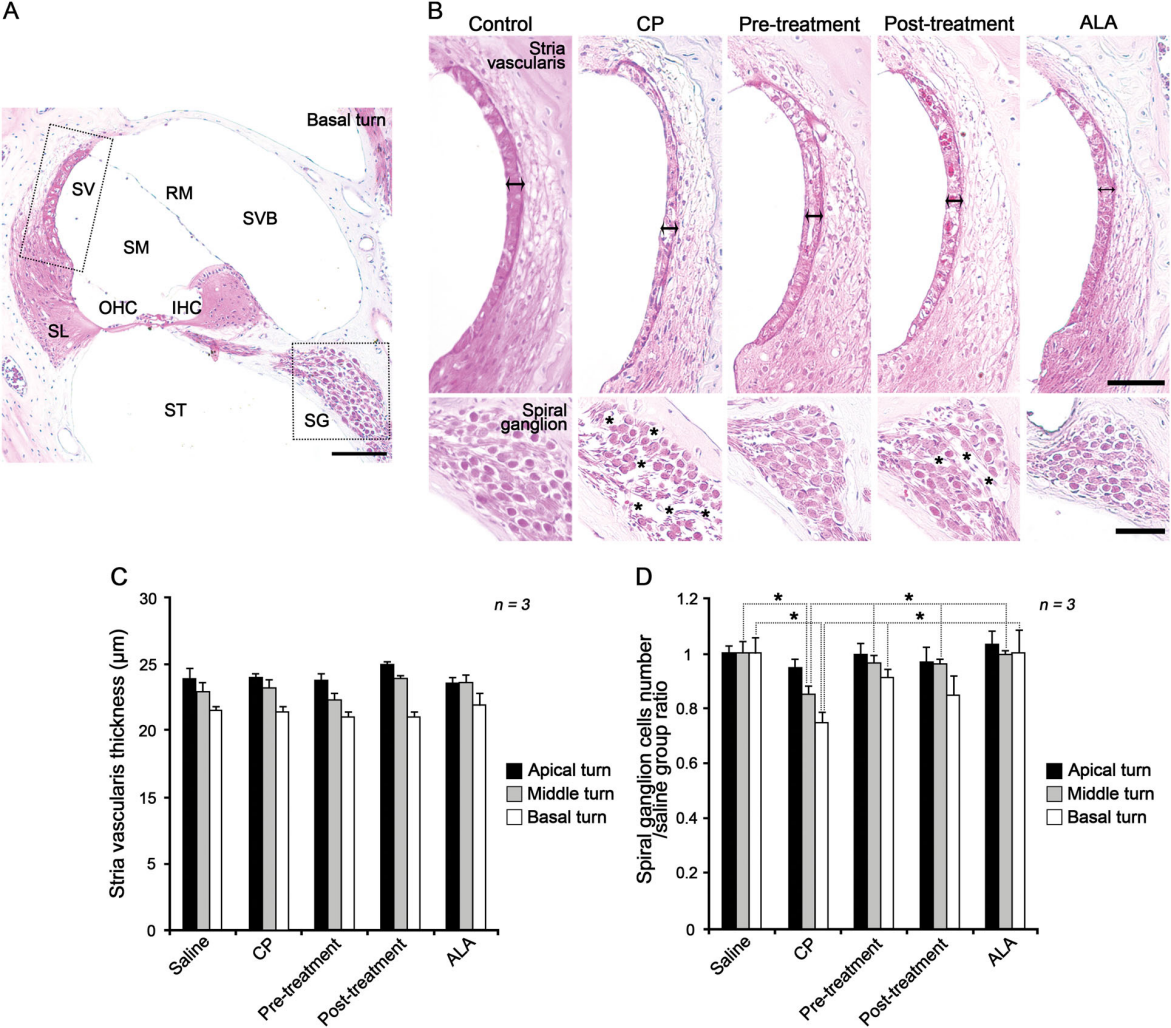
Histological analysis of the inner ears from cisplatin-injected mice receiving pre- or post-treatment with ALA. Hematoxylin and eosin staining was performed on inner ear sections from mice of each group (*n* *=* 3). **a** Basal turn of the inner ear from a mouse of the control group. **b** High magnification of stria vascularis (upper) and spiral ganglion (lower). Double arrows indicate thickness of stria vascularis and asterisks indicate degeneration of spiral ganglion cells. Scale bars represent 50 µm. **c** Quantitative comparison of stria vascularis thickness and **d** relative comparison of the number of spiral ganglion cells among each group at the basal, middle, and apical turns. The data are shown as the means ± SEM. *p < 0.05, compared with CP group. ALA alpha-lipoic acid, CP cisplatin, IHC inner hair cell, OHC outer hair cell, RM Reissner’s membrane, SG spiral ganglion, SL stria ligament, SM scala media, ST scala tympani, SV stria vascularis, SVB scala vestibuli

Histological analysis using hematoxylin and eosin staining was performed to determine whether these changes in hearing ability were associated with morphologic abnormalities. Because the stria vascularis and spiral ganglion are considered to be the primary targets of cisplatin-induced ototoxicity, we focused on these two regions (Fig. 2a). In the stria vascularis, no noticeable histological differences were observed, including thickness between the control and all other groups (Fig. 2b (upper) and c). In the cochlea, the most severe cisplatin-induced damage was detected in the spiral ganglion, where the numbers of cells were 70% lower in basal turn of the cisplatin group than in the control group. By contrast, the number of spiral ganglion cells in the ALA pre- and post-treatment groups remained similar to that in the control group (Fig. 2b (lower) and d). These results were highly consistent with ABR threshold results, suggesting that the loss of spiral ganglion cells may directly cause cisplatin-induced hearing loss in this mouse model.

**Fig. 3:**
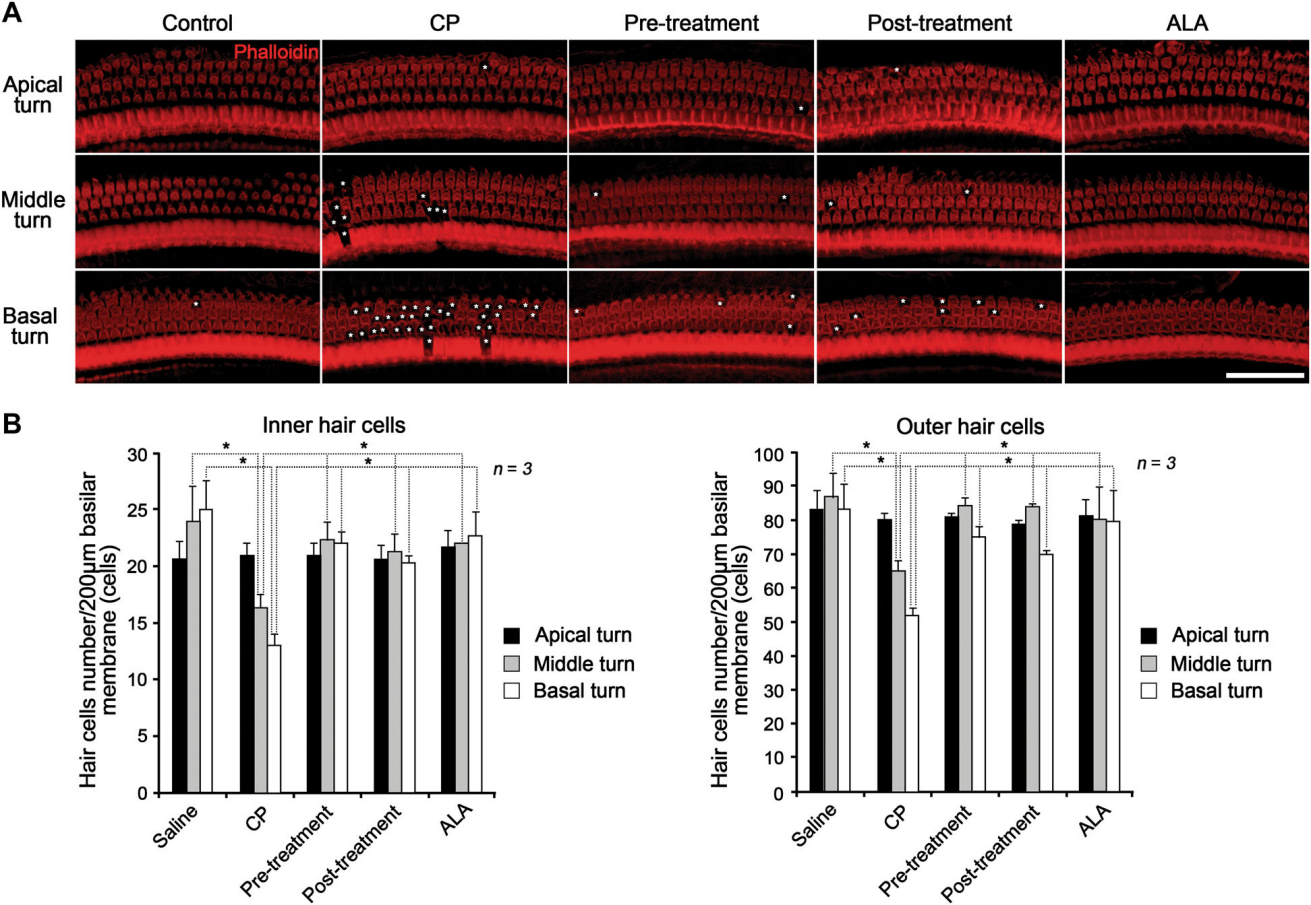
Effects of pre- and post-treatment with ALA on cisplatin-induced hair cell damage in cisplatin-injected mice. Protective and alleviative effects of ALA against ototoxicity in hair cells were confirmed by wholemount staining of the organ of Corti. **a** Phalloidin staining (red) of the organ of Corti including inner and outer hair cells to verify hair cell survival or degeneration. White asterisks indicate degenerating hair cells. The scale bar represents 50 µm. **b** Histogram quantitation of the number of inner and outer cells in each animal group at basal, middle, and apical turns of the cochlea. The data are shown as the means ± SEM. *p < 0.05, compared with CP group. ALA alpha-lipoic acid, CP cisplatin

Damage of the hair cells was examined using whole mount phalloidin staining of the organ of Corti to determine whether the hair cells were affected by cisplatin-induced ototoxicity. As a result, we found significant degeneration of inner and outer hair cells caused by cisplatin administration. Especially the area of the basal turn showed more severe damage than the areas of the middle and apical turns, and outer hair cells were more susceptible to cisplatin than inner hair cells (Fig. 3a). However, this severe damage was remarkably protected or alleviated by pre- or post-treatment with ALA (Fig. 3b).

### ALA reduces cisplatin-induced apoptotic cell death in HEI-OC1 cells

**Fig. 4:**
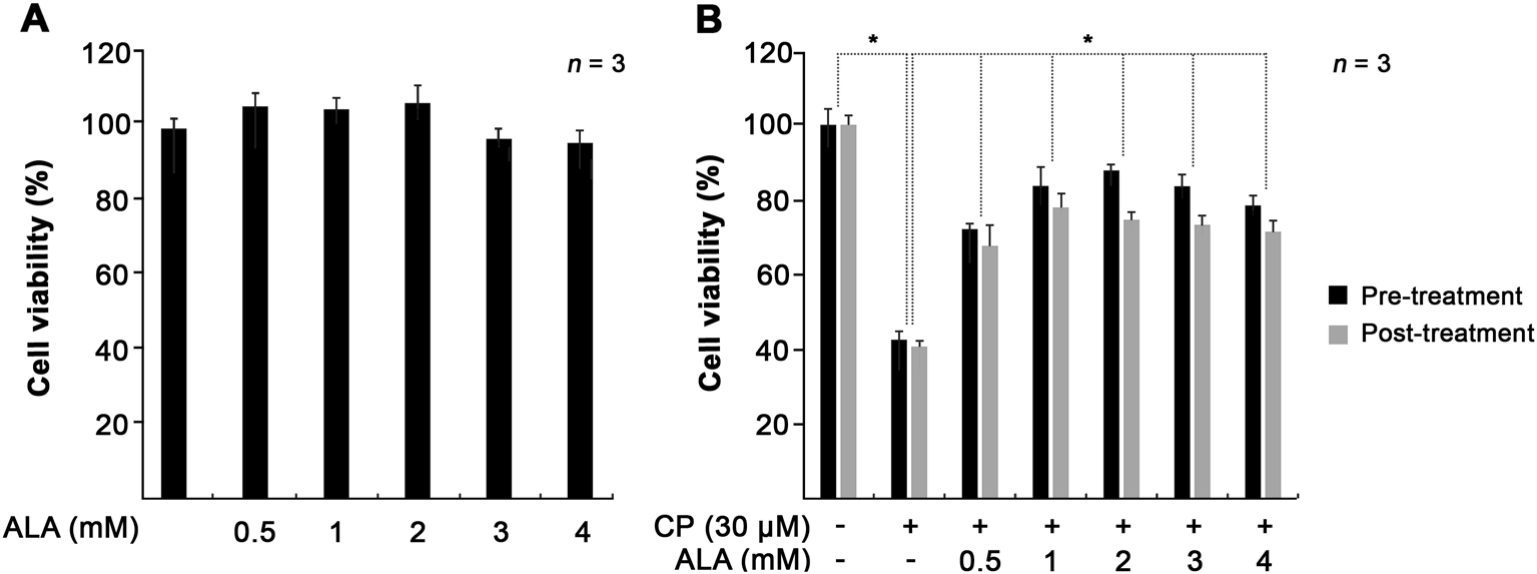
Effect of ALA treatment on cell viability in cisplatin-treated HEI-OC1 cells. **a** Cells were cultured with 0.5, 1, 2, 3, and 4 mM of ALA for 30 h, and cytotoxicity was evaluated using the MTT assay. **b** The cells were treated with 0.5−4 mM of ALA for 1 h, before or after treatment of 30 μM cisplatin for 30 h. The data represent the means ± SE of three separate experiments. *p < 0.05, compared with the CP group. ALA alpha-lipoic acid, CP cisplatin, HEI-OC1 House Ear Institute–Organ of Corti 1

To determine how pre- and post-treatment with ALA affects cisplatin-induced cytotoxicity, we investigated intracellular mechanisms controlled by ALA using House Ear Institute–Organ of Corti 1 (HEI-OC1) cells. Before this investigation, we first tested the cytotoxicity of ALA by measuring cell viability of untreated and ALA-treated cells. At various concentrations of ALA (0.5–4 mM), no cytotoxic effect on cell viability was observed (Fig. 4a). Treatment with cisplatin only decreased cell viability to 40%. However, with pre- or post-treatment of ALA, cell viability was significantly increased (Fig. 4b); the highest cell viability, 87%, was identified when cells were pretreated with 2 mM ALA. In addition, post-treatment of ALA significantly alleviated cell damage caused by cisplatin, reaching as much as 78% cell viability at 1 mM ALA. Considering these results and a previous study suggesting the toxic effects of 2 mM ALA by hair cell row disruption^19^, we used 1 mM ALA in the next experiments.

**Fig. 5:**
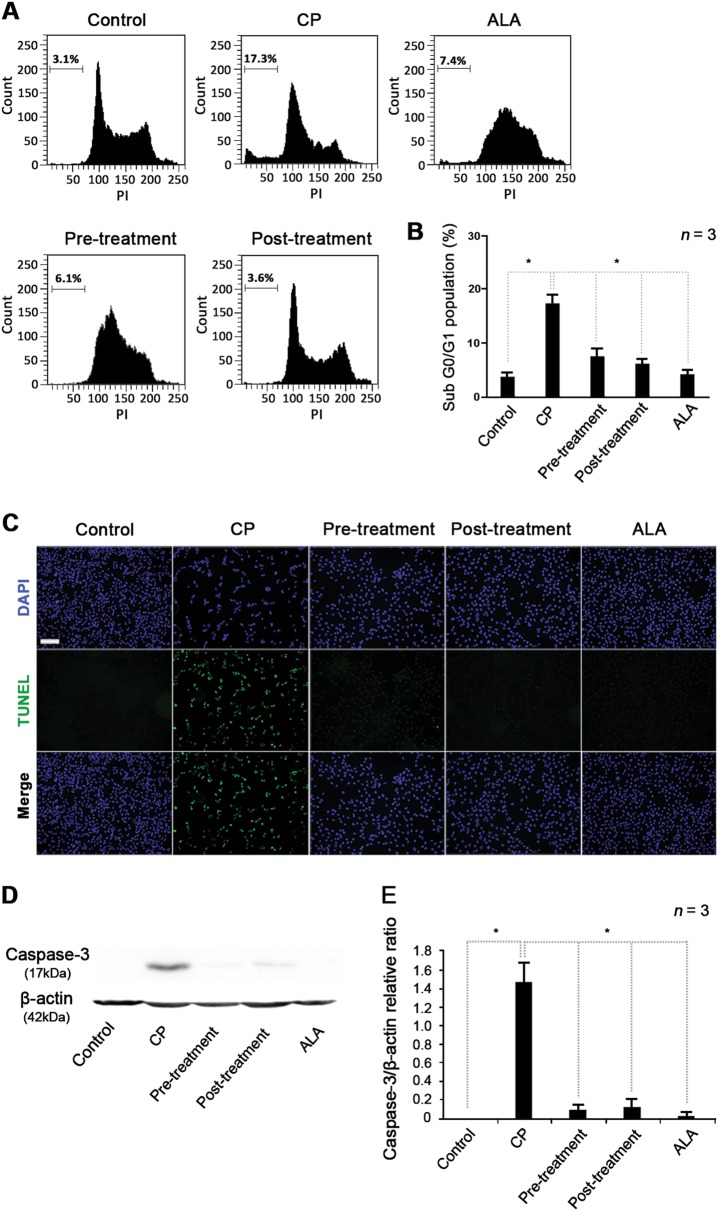
Effects of ALA on cell cycle arrest and apoptosis in cisplatin-treated HEI-OC1 cells. **a** Cell cycle analysis by flow cytometry and **b** comparison of the sub-G0/G1 ratio cell population between the CP group and ALA treatment groups. **c** TUNEL assay to detect apoptotic cells among treatment groups. Fragmentation of nucleic DNA (green) and the nuclei (blue) were stained and observed under a fluorescence microscope. The scale bar represents 100 µm. **d** Western blot of caspase-3 expression in cells treated with cisplatin and ALA. **e** Relative changes in caspase-3 expression were measured using densitometry by caspase-3:β-actin ratio. The cells were pre- or post-treated with 1 mM of ALA for 1 h, and 30 μM cisplatin for 30 h. The data are shown as the means ± SD. *p < 0.05, compared with CP group. ALA alpha-lipoic acid, CP cisplatin, DAPI 4′, 6-diamidino-2-phenylindole, HEI-OC1 House Ear Institute–Organ of Corti 1, TUNEL terminal deoxynucleotidyl transferase dUTP nick-end labeling

The protective role of ALA against cisplatin-induced cell death was examined by analysis of the cell cycle and apoptotic signaling pathway. Because previous studies have suggested that the cross-linking of cisplatin and DNA causes cell cycle arrest, we measured sub-G0/G1 fraction ratio using flow cytometry. We found that the cisplatin group had a larger cell population in the sub-G0/G1 phase than the control group (17 vs. 4%). However, the pre- and post-ALA treatment groups had only 6–7% of their cell populations in this phase (Fig. 5a, b). These results suggest that ALA can protect the cells from and alleviate cisplatin-induced effects on cell cycle arrest. In the cisplatin group, the TUNEL assay showed a high signal that was not seen in the control group. By contrast, only minimal TUNEL signals were detected in the ALA pre- and post-treatment groups, indicating substantially lower signal intensity than the cisplatin group (Fig. 5c). In addition, the ability of ALA to lower apoptotic signals was observed by caspase-3 expression analysis. In cisplatin-treated cells, strong caspase-3 expression was detected by western blot; however, the control group showed no visible expression. By contrast, pre- and post-treatment with ALA mostly inhibited caspase-3 expression (Fig. 5d, e). These results suggest that pre-treatment with ALA can protect cells against cisplatin cytotoxicity, and post-treatment with ALA can alleviate cell death by inactivation of the cisplatin-induced, caspase-3-dependent apoptosis signaling pathway.

### ALA effectively reduces ROS levels in HEI-OC1 cells

**Fig. 6:**
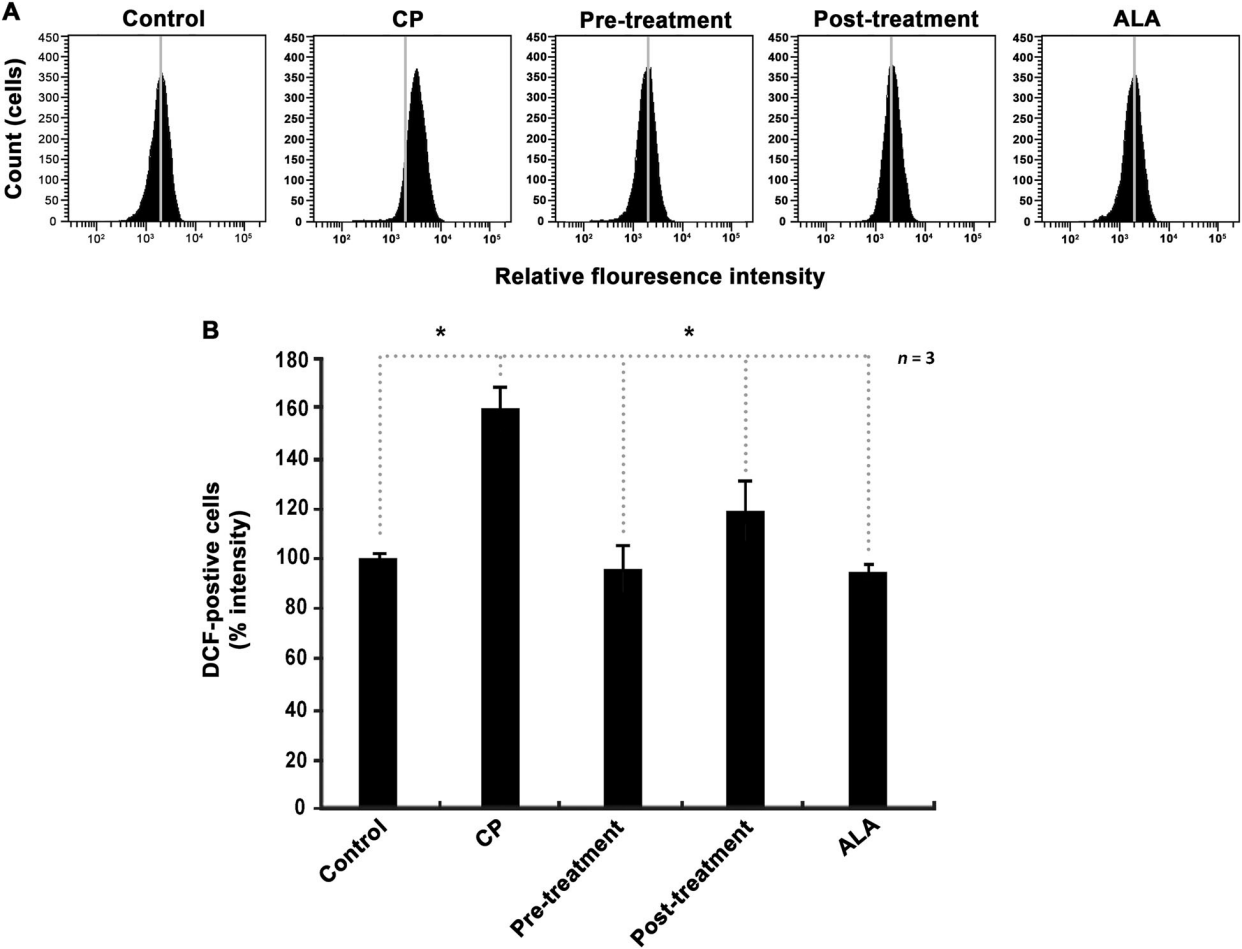
ROS scavenging capacity of ALA in cisplatin-treated cells. **a** Measurement of intracellular ROS level using DCFH-DA probe. The fluorescence intensity was detected by using a flow cytometer. **b** Relative FACS fluorescence intensities. The cells were cultured with 1 mM ALA for 1 h, before or after 30 μM cisplatin treatment for 24 h. ALA alpha-lipoic acid, CP cisplatin. The data are shown as the means ± SD. *p < 0.05, compared with CP group

One of the major triggers of cisplatin-induced apoptosis is excessive accumulation of intracellular ROS, and ALA is well-known as an effective antioxidant. Thus, we performed the 2′, 7′-dichlorofluorescein diacetate (DCFH-DA) assay to determine whether ALA reduces intracellular ROS generated by cisplatin in HEI-OC1 cells. The cisplatin group showed an approximately 1.6-fold greater fluorescent intensity than the control group, indicating a cisplatin-induced increase in ROS levels. By contrast, the ALA pre-treatment group maintained fluorescent intensity similar to the control group. In addition, the ALA post-treatment group had significantly less fluorescence intensity than the cisplatin group (Fig. 6a, b). These results indicate that ALA reduces intracellular ROS level overaccumulated after cisplatin treatment, as well as it prevents cisplatin-induced ROS generation before treatment.

**Fig. 7:**
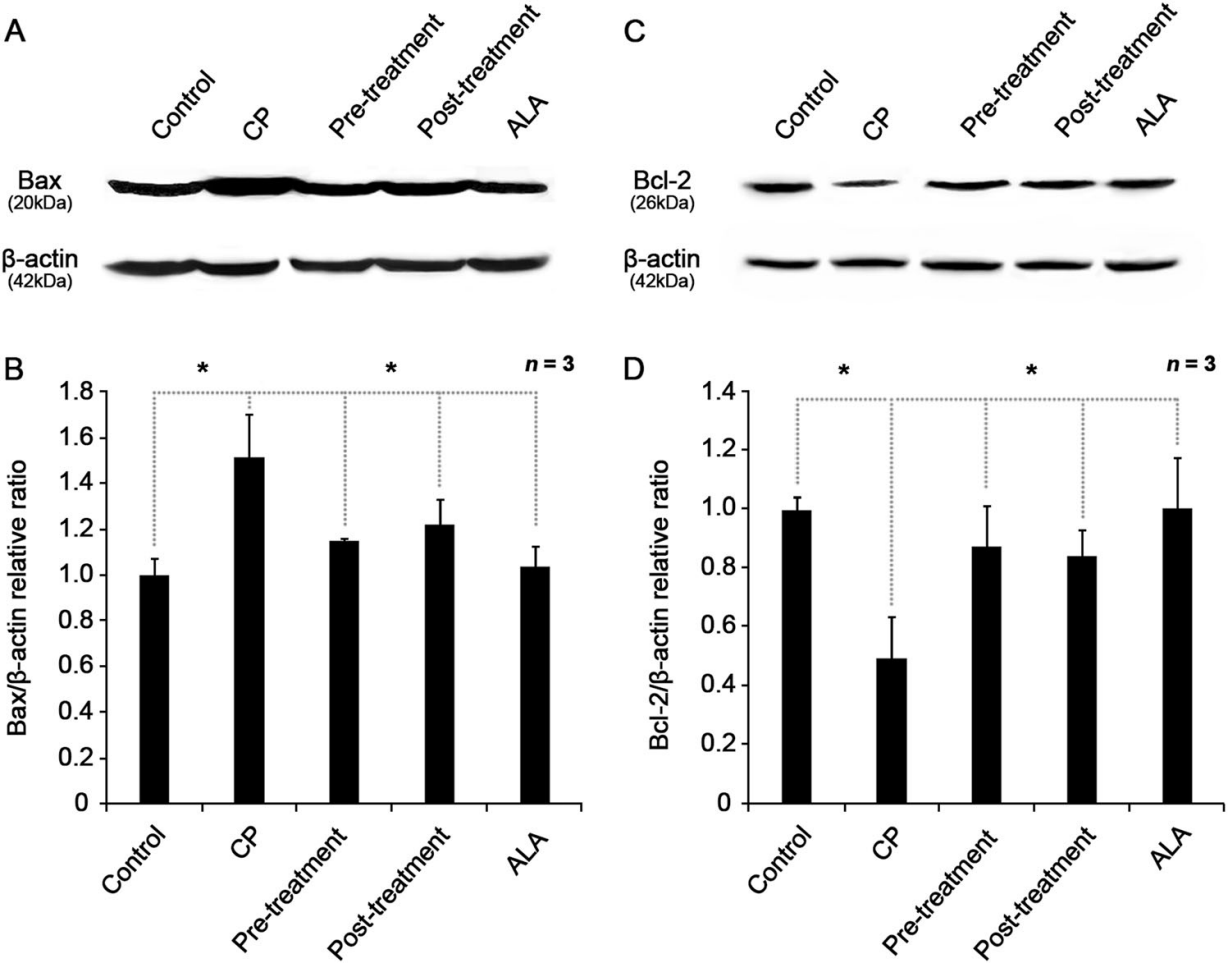
Antiapoptotic effect of ALA on the Bax-mediated apoptosis caused by cisplatin. **a** Western blot analysis of Bax expression, compared with β-actin as the loading control (*n* *=* 3 per lane). **b** Relative expression ratio of Bax and β-actin measured by densitometry. Cisplatin treatment upregulated Bax expression more than the control, and ALA-treated cells significantly alleviated Bax expression. **c** Western blot analysis of Bcl-2 expression, compared with β-actin as the loading control (*n* *=* 3 per lane). **d** Relative changes in Bcl-2 expression were measured using densitometry by Bcl-2:β-actin ratio. Cisplatin treatment downregulated Bcl-2 expression, and ALA-treated cells restored Bcl-2 expression. Cells were incubated with 1 mM ALA for 1 h, and/or 30 μM cisplatin for 24 h. The data are shown as the means ± SEM. *p < 0.05, compared with CP group

ROS scavenging of ALA was shown to eventually inhibit apoptosis through regulation of proapoptotic protein Bax and antiapoptotic protein Bcl-2. Using western blot analysis, we found that increased Bax expression (Fig. 7a, b) and decreased Bcl-2 expression (Fig. 7c, d) caused by cisplatin treatment was recovered by pre- or post-ALA treatments. Bax that is present in the cytoplasm migrates to the mitochondrial intermembrane space following oxidative stress, where it induces apoptosis by releasing cytochrome *c*. Bcl-2 inhibits Bax activity to suppress apoptosis and promote cell survival^20^. Therefore, ROS accumulation increased by cisplatin induces Bax to cause mitochondrial dysfunction leading apoptosis, and ALA plays a central role in protecting mitochondria from this cisplatin-induced oxidative stress. Importantly, although the cells were already damaged by cisplatin in the ALA post-treatment group, ALA successfully inhibited Bax expression, leading to less apoptosis of the affected cells.

**Fig. 8:**
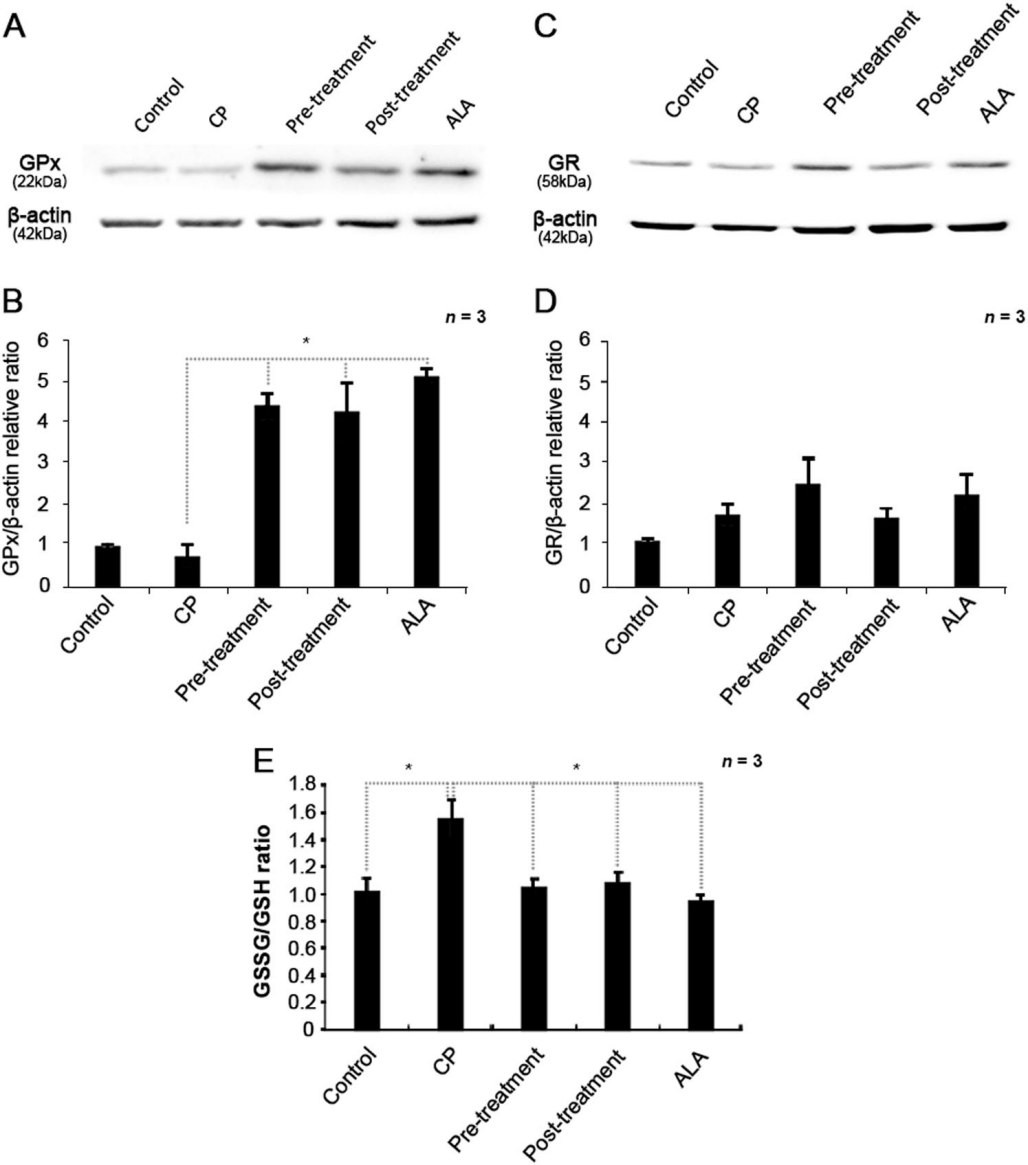
Expression levels of antioxidant enzymes GPx and GR in cisplatin-treated cells receiving pre- or post-treatment with ALA. **a** Western blot of GPx expression, compared to β-actin as a loading control (*n* *=* 3 per lane). **b** Relative expression ratio of GPx and β-actin measured by densitometry. ALA treatment upregulated the expression of GPx more than the control treatment. **c** Western blot of GR expression, compared to β-actin as a loading control (*n* *=* 3 per lane). **d** Relative expression ratio of GR and β-actin measured by densitometry. Increases in GR expression by ALA treatments were not significant. **e** The ratio of GSH to GSSG normalized to total protein content. Cells were treated with 1 mM ALA and 30 μM cisplatin for 1 and 24 h, respectively. Normalized data are presented as the means ± SD. ALA alpha-lipoic acid, CP cisplatin, GPx glutathione peroxidase, GR glutathione reductase, GSH glutathione, GSSG glutathione disulfide. *p < 0.05, compared with CP group

ALA is reportedly involved in the oxidation and reduction of glutathione, a representative antioxidant. GSH, a reduced form of glutathione, is oxidized to Glutathione disulfide (GSSG) by glutathione peroxidase (GPx), resulting in a decrease of cellular ROS, and GSSG is reduced to GSH by glutathione reductase (GR). Because this glutathione redox cycle is known to play a key role in reducing the oxidized form of GSSG to GSH, we examined expression levels of GPx and GR by western blots. Surprisingly, GPx expression was increased by 4- to 5-fold in all groups treated with ALA (Fig. 8a, b), suggesting that ALA-induced GPx expression can actively remove ROS. Interestingly, the increase in GR expression was not significant compared to GPx expression (Fig. 8c, d), which indicates that increased GSSG could not be reduced to GSH due to insufficient expression of GR. However, the GSSG/GSH ratios of both ALA pre- and post-treatment groups remained similar to that of the control group, even though GR expression was not significantly increased (Fig. 8e). These data suggest that ALA may help to reduce ROS continuously, performing the role of GR to reduce GSSG to GSH.

## Discussion

In this study, we investigated the therapeutic effect of ALA in cisplatin-induced sensorineural hearing loss using in vitro and in vivo model systems. When treatment followed cisplatin administration, ALA showed a strong alleviative effect on cisplatin-induced ototoxicity, which eventually prevented major hearing loss in the mouse model. The intracellular mechanism affected by cisplatin was excessive accumulation of ROS, leading to mitochondrial dysfunctions, and ALA was found to restore the redox system by supporting GR activity.

Since cisplatin-induced hearing loss was first reported^9^, numerous studies have investigated the physiological features and mechanisms of ototoxicity caused by cisplatin. Cochlear damage due to cisplatin is primarily found in the organ of Corti, including the hair cells, spiral ganglions, and stria vascularis^10,21^. Consistent with previous studies, we found severe hair cell loss and degeneration of spiral ganglions, leading to sensorineural hearing loss. However, another major defect, atrophy of the stria vascularis due to a decrease in the intermediate cell and marginal cell area, was not significant, even though the stria vascularis has been previously considered to be a primary site of cisplatin damage^3^. Because various types of potassium channels and pumps in intermediated and marginal cells of the stria vascularis play an indispensable role in maintaining a positive endocochlear potential, reduction of this potential due to loss of these cells has been thought to lead to stria vascularis dysfunction, causing hair cell degeneration and eventually leading to hearing loss^22,23^. However, our data showed a significant reduction in the number of hair cells and spiral ganglions of the cisplatin-treated mouse cochlea without notable histological abnormalities in the stria vascularis. These findings suggest that the loss of hair cells and spiral ganglions observed in this study may be triggered by their own intracellular apoptotic signaling pathway, and not only by stria vascularis dysfunction.

Excessive accumulation of intracellular ROS has been recognized as the strongest trigger of cisplatin-induced initiation of caspase-3 and -9 activities, resulting in increased apoptosis^24^. As the most vulnerable organelle to oxidative stress, mitochondria are known to be the first target of cisplatin-induced ROS damage. Evaluating mitochondrial-dependent ROS response to cisplatin cytotoxicity, Marullo et al. showed that cells lacking functional mitochondria were more resistant to cisplatin-induced cell death^7^. In addition, cisplatin is known to deplete mitochondrial antioxidant enzyme activities, a finding that has been verified by cisplatin-induced increases in lipid peroxidation and GSSG/GSH ratio^25–28^. In the organ of Corti, several previous studies found mitochondrial damage in cisplatin-affected cells and the spiral ganglion by detecting mitochondrial membrane permeabilization and cytoplasmic release of cytochrome *c*, which activates caspase-9 and -3^7,24,29^. This ROS-associated mitochondrial damage was consistently observed in our study. Increased expression of Bax (proapoptotic) and decreased expression of Bcl-2 (antiapoptotic), as well as increased ROS levels, indicated destructive change of mitochondrial membrane permeability^30^. Therefore, we expected that reducing mitochondrial ROS would protect cells from cisplatin-induced apoptosis.

Antiapoptotic effects of ALA, one of the most effective antioxidants, have been broadly demonstrated in several cisplatin-induced damages, including nephrotoxicity, neurotoxicity, and ototoxicity^31,32^. Several functional studies on ototoxicity have found that pretreating with ALA before exposure to cisplatin can significantly prevent ROS accumulation in cochlear cells and protect hearing function^31^. In the cellular antioxidant system, ALA is known to contribute to the recycling of GSSG and reduced GSH, a process that plays a key role in ROS regulation to protect cells from oxidative stress^33^. By helping to reduce GSSG to GSH, levels of intracellular GSH are maintained, enabling its use as a substrate of GPx to reduce hydrogen peroxide to water^34^. In this study, we also identified antioxidative effect of ALA through rescued Bcl-2 and Bax expression and GSSG/GSH ratio in both pre- and post-treatment of ALA. Moreover, GPx expression was highly upregulated by ALA. Emphasizing the important role of GPx in the antioxidant system, a previous study showed that GPx affects the antiapoptotic Bcl-2 expression and its activity. GPx overexpression potently inhibited apoptotic signaling by increasing Bcl-2 expression and decreasing caspase-9 activity in cisplatin-treated H460 cells, and its antiapoptotic effect was much stronger than that of another antioxidant enzyme, superoxide dismutase^35^. Therefore, ALA has a central role in protecting cells from ROS-induced apoptotic cell death through the efficient upregulation of GPx^36^. By contrast, increased activity of GR, an enzyme that produces GSH by reducing GSSG, was relatively insignificant in our study, which corresponds with previous ALA studies^36,37^. In these studies, ALA showed no influence on the activity of GR; however, it remarkably increased GPx activity in rat blood, liver, kidney, and heart. Considering that ALA treatment reduced GSSG/GSH ratio (despite the nonsignificant increase in GR activity compared to highly upregulated GPx expression), we suggest a possibility that ALA directly assists or partially substitutes for GR function in the GSH−GSSG redox reaction. This study provides direct evidence that ALA plays a specific role in the antioxidant system against oxidative stress, and strongly suggests a therapeutic role of ALA in cisplatin-induced ototoxicity as a GSSG reductant.

To our knowledge, this is the first report to demonstrate a therapeutic effect of ALA in cisplatin-induced ototoxicity using both in vitro and in vivo systems. So far, most ototoxicity studies have only focused on the protective effect of ALA on cisplatin-induced hearing loss, using it as a pretreatment before cisplatin administration. However, because many patients already suffer from hearing damage after cisplatin cancer therapy, it is very important to find effective medications that have a therapeutic effect on existing cisplatin-induced ototoxicity. This study directly showed the quantitative recovery of intracellular Bax and Bcl-2 levels and GSSG/GSH ratio in HEI-OC1 cells, as well as the protection of hearing ability in a mouse model, with ALA post-treatment after cisplatin administration. It means that ALA can effectively restore mitochondrial redox system resulting in inhibition of cisplatin-induced apoptotic cell death, which emphasize a strong significance of this study that post-treatment with ALA may provide a therapeutic option for patients with existing cisplatin-induced hearing loss. Furthermore, exploring other effective mitochondria-targeted nutrients that can be coadministrated with ALA will be a highly valuable step to maximize the antioxidative effect of ALA. Coadministration of vitamin E and acetyl-l-carnitine with ALA was reported to produce a synergistic effect^38–41^, and other antioxidants or mitochondrial enzyme cofactors were also applied with ALA to prevent cell aging in other studies^41,42^. Thus, identification of an ideal administration partner of ALA will be critical to achieve effective and safe clinical application of ALA to rescue auditory systems damaged by cisplatin.

In summary, this is the first study to report on a strong therapeutic potential of ALA to rescue ototoxic hearing loss caused by cisplatin, and our data provide key evidence that ALA may act as a reducing agent for GSSG to increase GSH levels on behalf of GR. Our results were consistent in cultured cells and an animal model, which improves the clinical value of ALA for therapy of cisplatin-induced ototoxicity. Further comprehensive research on coadministration of various reagents that affect different intracellular pathways and its effective intracochlear delivery systems will provide us with an important foundation to overcome ototoxic hearing loss.

## Materials and methods

### Animals

Male and female mice of the Institute for Cancer Research strain (8 weeks old) were purchased from Hyochang Science (Daegu, Republic of Korea). All animal procedures were conducted in accordance with the Institutional Animal Care guidelines issued by the Committee of Animal Research at Kyungpook National University.

### In vivo experimental design

Mice were divided into five groups: control group, cisplatin group, ALA pretreatment group, ALA post-treatment group, and ALA group. In all these groups, mice were injected with 20 mg/kg of cisplatin (Sigma, St. Louis, MO, USA) and/or 100 mg/kg of ALA (Thioctacid inj^®^, Bukwang Pharmaceutical Co., Ltd., Seoul, Korea) by intraperitoneal administration. For the groups that received cisplatin, ABR threshold was measured before injections and 4.5 days after cisplatin injection to evaluate changes in hearing ability, as previously described (Supplementary Figure 1). The control group was treated with 0.9% saline only for 3 days. The ALA pretreatment group was injected with ALA for 2 days, followed by cisplatin administration. The ALA post-treatment group was first injected with cisplatin, and then received ALA for the next 2 days. The ALA group was injected with ALA only for 3 days.

### Measurement of auditory brainstem response

To assess auditory function, we performed ABR measurements using an ABR workstation (System 3, Tucker Davis Technology, Inc., Alachua, FL, USA). All tests were conducted in a soundproof room. Briefly, prior to ABR measurement, animals were anesthetized by intramuscular injection of alfaxalone (40 mg/kg) and placed on a heating pad to maintain their body temperature at 37 °C. Mouse body temperature was monitored using a rectal thermometer. To record the ABRs, subcutaneous needle electrodes were inserted into the vertex (+charge), mastoid (−e), and hind leg (ground). Acoustic stimuli, consisting of either a tone-burst stimulus with a 1-ms rise/fall time and a 5-ms plateau at frequencies of 8, 16, and 32 kHz or transient click stimuli, were applied monaurally through a speaker. The stimulus signals were generated using a SigGenRP and an RP2.1 real-time processor, and then transmitted through a programmable attenuator (PA5, TDT), a speaker driver (ED1, TDT), and an electrostatic speaker (EC1, TDT). Stimuli were generated for 500 repetitions in 5-dB decrements, starting from a 90-dB sound pressure level to the acoustic threshold at every frequency. The phase of the stimulus was reversed upon each presentation to reduce the artifacts caused by repetitive stimuli.

### Paraffin sectioning and histological analysis

Mice were perfused with 1× phosphate-buffered saline (PBS), and the inner ears were isolated. For paraffin sections, the inner ears were fixed with 4% paraformaldehyde (PFA) in PBS for 24 h at 4 °C, and then decalcified in 10% ethylenediaminetetraacetic acid in PBS for another 24 h at 4 °C. The inner ears were dehydrated with a graded ethanol series, permeabilized with xylene, and embedded in paraffin at room temperature. The paraffin-embedded inner ears were then serially sectioned into 6-μm-thick slices using a microtome (Leica RM2235, Leica Microsystems, Germany) and mounted on Superfrost Plus microscope slides (Fisher Scientific, Pittsburgh, PA, USA) for staining. All slides were maintained at 4 °C until use. The slides with paraffin-embedded inner ear sections were incubated for 1 h at 65 °C, deparaffinized with xylene, and rehydrated using a graded ethanol series. Nuclei and cytoplasm were stained with hematoxylin and eosin reagent (Sigma). To verify the density of spiral ganglion cells, we determined the percentage of cells by counting the average number of spiral ganglion cells in a 1 mm^2^ area of paraffin section in each turn (basal, middle, and apical) of the cochlea.

### Phalloidin staining

The cochlea was fixed with 4% PFA in PBS at room temperature for 20 min, washed with 1× PBS, and incubated with 0.1% Triton X-100 at room temperature for 15 min. The organ of Corti was dissected from the cochlea, stained with Alexa Fluor 555 phalloidin (Invitrogen-Molecular Probes, Eugene, OR, USA, 1:1000) in PBS for 2 h in the dark, and washed three times with PBS. The organ of Corti was observed in whole mounts using a confocal microscope (LSM 700; Zeiss, Oberkochen, Germany) to count the average number of normal hair cells in a 200 μm region of each turn of the cochlea.

### Cell culture and viability assay

HEI-OC1 auditory cells were cultured under permissive conditions (33 °C, 10% CO_2_) for 16 h in Dulbecco’s modified Eagle’s medium (Hyclone, Logan, UT, USA) containing 10% fetal bovine serum (Hyclone, Logan, UT, USA) and 50 units/mL IFN-γ (PeproTech, EC, London, UK) without antibiotics. In all of experiments in vitro, each group of cells (7 × 10^3^ cells/well of 96-well plate) were pre- or post-treated with 1 mM ALA for 1 h, before or after 30 μM cisplatin treatment for 30 h. Cell viability was measured using a 3-(4,5-dimethylthiazol-2-yl)-2,5-diphenyltetrazolium bromide (MTT; Sigma, St. Louis, MO, USA). After the cells were treated with 0.5, 1, 2, 3, 4 mM of ALA and/or 30 μΜ cisplatin for 30 h, 0.5 mg/mL of MTT solution was added to cell culture media, followed by incubation for 2 h at 33 °C with 10% CO_2_. The optical density of each well was measured using a microplate reader at 550 nm.

### Measurement of intracellular ROS production

HEI-OC1 cells were cultured for 16 h, and then treated with 30 μM cisplatin for 24 h in the presence or absence of ALA pre- and post-treatment. Intracellular ROS levels were measured using the fluorescent dye, DCFH-DA (Invitrogen-Molecular Probes, Eugene, OR, USA). Cells were washed twice with 1× PBS and incubated with 10 μM DCF-DA for 5 min at 33 °C and 10% CO_2_. Then, flow cytometry analyses (10,000 events per sample) of DCFH-DA incubated cells were performed using a BD FACS Aria III flow cytometer (BD Biosciences, San Diego, CA, USA).

### Cell cycle analysis

HEI-OC1 cells were trypsinized, counted, centrifuged, and fixed in 70% ethanol at 30 h after 30 μΜ cisplatin treatments. The cells were washed twice with 1× PBS and centrifuged. The centrifuged pellets were resuspended in a solution of RNase A (0.2 mg/mL) and propidium iodide (10 μg/mL) and incubated at 4 °C for 30 min. Cell cycle distribution was determined using a BD FACS Aria III flow cytometer (BD Biosciences, San Diego, CA, USA).

### Detection of DNA fragmentation

Cell apoptosis was analyzed using an in situ cell death detection kit (Roche Biochemicals, Mannheim, Germany) based on the terminal TUNEL technique. HEI-OC1 cells were cultured on a 24-well culture plate and treated with cisplatin for 30 h. Then, cells of each experimental group were fixed in 4% PFA for 15 min at room temperature, then washed and permeabilized for 5 min at 4 °C with freshly prepared 0.1% Triton X-100 and 0.1% sodium citrate in water. TUNEL reaction mixture (50 μL) was added to the samples and incubated for 60 min at 37 °C, protected from light. All samples were washed three times with 1× PBS for 5 min each. Then, nuclei were stained with 4′, 6-diamidino-2-phenylindole for 5 min. Specimens were visualized using fluorescence microscopy (Carl Zeiss, Oberkochen, Germany).

### Protein preparation

HEI-OC1 cells were washed with ice-cold 1× PBS and suspended in 100 μL RIPA buffer (Elpis Biotech, Daejeon, Korea) containing a cocktail of protease inhibitors (Calbiochem Protease Inhibitor Cocktail Set 1, La Jolla, CA, USA). The suspension was transferred into a pre-cooled 1.5 mL tube, incubated on ice for 15 min, centrifuged at 13,000 rpm for 30 min at 4 °C, and vortexed for 1 min. The supernatant was aspirated and placed in a fresh tube on ice, and the pellet was discarded.

### Western blot analysis

HEI-OC1 cell extracts were analyzed by 12% sodium dodecyl sulfate–polyacrylamide gel electrophoresis. The separated proteins were electrotransferred to a nitrocellulose membrane. Membranes were blocked with a solution of 20 mM Tris-HCl (pH 7.6), 137 mM NaCl, and 0.01% Tween-20 (TBS-T) containing 5% skim milk for 1 h, and then probed with primary antibodies (1:100–1:2000 dilution) at room temperature. Next, the membranes were washed three times for 5 min each with TBS-T and incubated with secondary antibody (1:2000 dilution). After a series of washes, the membranes were developed using an enhanced chemiluminescent detection system. Rabbit anti-Bax and anti–caspase-3 antibodies were from Cell Signaling (Cell Signaling, Danvers, MA, USA), and rabbit anti-Bcl-2, anti-GR, and anti-GPx antibodies were from Abcam (Abcam, Cambridge, MA, UK). Goat anti-rabbit-lgG-HRP was used as the secondary antibody (Cell Signaling, Danvers, MA, USA).

### Measuring glutathione levels

Cellular GSH levels were measured in HEI-OC1 cell lysates exposed to cisplatin for 24 h using a GSH colorimetric detection kit (BioVision Inc., Milpitas, CA, USA). Briefly, cells were scraped and suspended in 60 μL ice-cold glutathione buffer, and each sample was mixed with 20 μL perchloric acid. Samples were kept on ice for 5 min, and centrifuged at 13,000 rpm for 2 min at 4 °C. Supernatant was collected, and 20 μL ice-cold 6 N KOH solution was added to 40 μL of the resulting samples. These samples were kept on ice for 5 min and centrifuged in the cold. Next, 10 μL neutralized supernatant was transferred to a 96-well plate. To detect GSH, 80 μL assay buffer was added to each well. Total GSH samples (70 μL) was added to 10 μL reducing agent mix and incubated for 10 min. GSSG samples were added to 60 μL assay buffer and 10 μL GSH quencher for 10 min, and then 10 μL reducing agent was added, and the samples were incubated for 10 min. Finally, 10 μL o-phthalaldehyde was added to all samples and incubated for 40 min at room temperature.

### Statistical analyses

Data were analyzed using SPSS statistics software package (version 23; SPSS, Chicago, IL, USA). Continuous variables were expressed as mean ± standard deviation. One-way ANOVA was used to analyze the variables among the 5 or 6 groups. Student’s *t* test was used to analyze between the two groups. A *p* value of <0.05 was considered statistically significant.

## Electronic supplementary material

Supplementary Figure 1
