# Supplementary material for: Evaluating protective and therapeutic effects of alpha-lipoic acid on cisplatin-induced ototoxicity
Source: Cell Death Dis. 2018 Aug 1;9(8):827. doi: 10.1038/s41419-018-0888-z (PMC6070527; doi:10.1038/s41419-018-0888-z)
Supplement: Supplementary file 1 — Supplementary Figure 1 [file 41419_2018_888_MOESM1_ESM.docx]

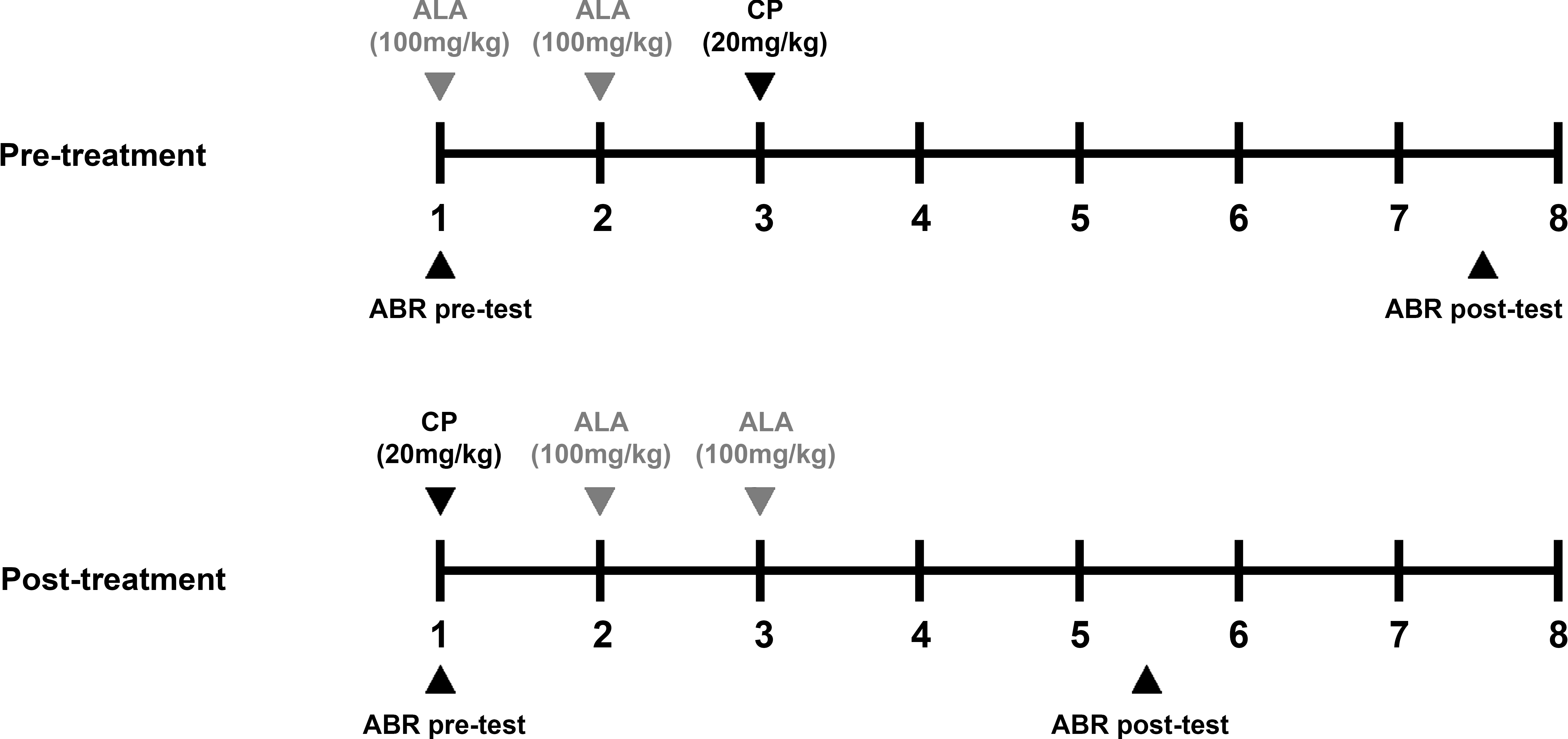


**Supplementary Figure 1. Schematic diagram of the cisplatin injection timeline for mice in the ALA pre- and post-treatment groups.** Mice received alpha-lipoic acid (ALA) injections either before or after cisplatin injections. Auditory brainstem response (ABR) was measured before injections (day 1) and 4.5 days after cisplatin injection (pre-treatment: day 7.5; post-treatment: day 5.5).
